# Interplay of Filaggrin Loss-of-Function Variants, Allergic Sensitization, and Eczema in a Longitudinal Study Covering Infancy to 18 Years of Age

**DOI:** 10.1371/journal.pone.0032721

**Published:** 2012-03-05

**Authors:** Ali H. Ziyab, Wilfried Karmaus, Mitra Yousefi, Susan Ewart, Eric Schauberger, John W. Holloway, Hongmei Zhang, Syed Hasan Arshad

**Affiliations:** 1 Department of Epidemiology and Biostatistics, Norman J. Arnold School of Public Health, University of South Carolina, Columbia, South Carolina, United States of America; 2 Department of Community Medicine and Behavioral Sciences, Faculty of Medicine, Kuwait University, Kuwait City, Kuwait; 3 College of Veterinary Medicine, Michigan State University, East Lansing, Michigan, United States of America; 4 Genetics Graduate Program, Michigan State University, East Lansing, Michigan, United States of America; 5 Academic Units of Clinical and Experimental Medicine, Faculty of Medicine, University of Southampton, Southampton, United Kingdom; 6 Academic Units of Human Genetics, Faculty of Medicine, University of Southampton, Southampton, United Kingdom; 7 David Hide Asthma and Allergy Research Centre, Isle of Wight, United Kingdom; 8 College of Osteopathic Medicine, Michigan State University, East Lansing, Michigan, United States of America; Leibniz-Institute for Arteriosclerosis Research at the University Muenster, Germany

## Abstract

**Background:**

Immune specific genes as well as genes regulating the formation of skin barrier are major determinants for eczema manifestation. There is a debate as to whether allergic sensitization and filaggrin gene (*FLG*) variants lead to eczema or *FLG* variants and eczema increase the risk of allergic sensitization. To investigate the time-order between eczema and allergic sensitization with respect to *FLG* variants, data from a large prospective study covering infancy to late adolescence were analyzed.

**Methodology/Principal Findings:**

Repeated measurements of eczema and allergic sensitization (documented by skin prick tests) at ages 1, 2, 4, 10, and 18 years were ascertained in the Isle of Wight birth cohort (n = 1,456). Three transition periods were analyzed: age 1-or-2 to 4, 4 to 10, and 10 to 18 years. *FLG* variants were genotyped in 1,150 participants. Over the three transition periods, in temporal sequence analyses of initially eczema-free participants, the combined effect of *FLG* variants and allergic sensitization showed a 2.92-fold (95% CI: 1.47–5.77) increased risk ratio (RR) of eczema in subsequent examinations. This overall risk was more pronounced at a younger age (transition period 1-or-2 to 4, RR = 6.47, 95% CI: 1.96–21.33). In contrast, *FLG* variants in combination with eczema showed a weaker, but significant, risk ratio for subsequent allergic sensitization only up to 10 years of age.

**Conclusions/Significance:**

Taking the time order into account, this prospective study demonstrates for the first time, that a combination of *FLG* variants and allergic sensitization increased the risk of eczema in subsequent years. Also *FLG* variants interacted with eczema and increased the risk of subsequent allergic sensitization, which, was limited to the younger age. Hence, early restoration of defective skin barrier could prevent allergic sensitization and subsequently reduce the risk of eczema development.

## Introduction

The prevalence of common allergic disorders including eczema has increased in recent decades among children in westernized societies [1]. Eczema is a common chronic inflammatory skin disorder characterized by relapsing-remitting combination of skin dryness, pruritus, and rash starting in childhood and often persisting into adulthood [1], [2]. Allergic sensitization, characterized by increased immunoglobulin-E (IgE) antibodies in response to specific allergens, has been linked to the increased risk of allergic disorders and is believed to account for 30% to 40% of cases of eczema, asthma, and allergic rhinitis [3], [4]. In past decades, most of the clinical etiology of eczema was attributed to genetic predisposition to sensitization, thus relating eczema to immune specific genes. However, the recently recognized role of a dysfunctional skin barrier in the pathogenesis of eczema suggests that a functional skin barrier could prevent the development of allergic sensitization and consequently eczema [5].

One important gene involved in the formation of a normal skin barrier is filaggrin (*FLG*). This gene encodes filament-aggregating protein, which serves as a primary shield against increased transepidermal water loss and entry of microbes, allergens, and irritants [5], [6]. The *FLG* gene is situated within the epidermal differentiation complex on chromosome 1q21. Null (Loss-of-function) variants in the *FLG* gene have been linked to disrupted skin barrier and are strong predisposing factors in the development of eczema [7]. In European populations, R501X and 2282del4 are the most prevalent *FLG* variants; their association with eczema has been replicated in several investigations, summarized in meta-analysis [8]. Three other less common *FLG* variants (3702delG, R2447X, S3247X) have also been associated with eczema [9], [10], [11]. It is estimated that about 10% of European populations carry at least one of the *FLG* variants [12]. Several studies have shown that *FLG* variants are major risk factors for eczema in general as well as for the subgroups of atopic and non-atopic eczema [13], [14], [15]. In addition, a meta-analysis study summarized findings of several investigations that *FLG* variants also increase the risk for allergic sensitization [16].

A complex interplay between genetic, environmental, and immunological factors is considered to contribute to the pathogenesis of eczema [17]. However, the time-order of the associations between allergic sensitization and eczema is highly disputed in the literature [18] without consensus on which comes first [19]. To our knowledge, analyses of longitudinal studies investigating the temporal sequence of the occurrence of allergic sensitization and eczema while accounting for the effect of *FLG* variants are lacking. One position is that allergic sensitization interacts with *FLG* variants leading to increased risk of eczema. The other position is that eczema interacts with *FLG* variants leading to higher risk of allergic sensitization [19]. This dispute is more than an academic discourse, since prophylaxis can either focus on avoiding allergic sensitization or preventing eczema. To solve this dispute, longitudinal studies are needed that can address the critical time order. Therefore, we assessed the interplay of *FLG* variants, allergic sensitization, and eczema in a longitudinal study.

The determination of *FLG* variants and repeated assessments of eczema and allergic sensitization at ages 1, 2, 4, 10 and 18 years in the Isle of Wight birth cohort facilitate the investigation of the combined effects. First, we investigated the individual and combined effects of allergic sensitization and *FLG* variants on the occurrence of eczema in a concurrent model (all manifestations were detected at the same age). Second, to elucidate the temporal sequence of events we tested two hypotheses: (i) the combined effect of eczema and *FLG* variants increases the risk of subsequent allergic sensitization and (ii) the combined effect of allergic sensitization and *FLG* variants increases the risk of subsequent eczema.

## Methods

### The Isle of Wight birth cohort

A whole population birth cohort was established on the Isle of Wight, UK, in 1989 to prospectively study the natural history of allergies from birth to 18 years of age. The island is close to the British mainland, semi-rural, without heavy industry. Both the Isle of Wight and the study populations are 99% Caucasian. Ethics approvals were obtained from the Isle of Wight Local Research Ethics Committee (now named the National Research Ethics Service, NRES Committee South Central – Southampton B) at recruitment and for the 1, 2, 4, 10 and 18 years follow-up (06/Q1701/34). Of the 1,536 children born between January 1, 1989, and February 28, 1990, written informed consent was obtained from parents to enrol 1,456 newborns. Children were followed up at the ages of 1 (n = 1,167), 2 (n = 1,174), 4 (n = 1,218), 10 (n = 1,373), and 18 years (n = 1,313). Detailed questionnaires were completed for each child at each follow-up. When a visit was not possible, a telephone questionnaire was completed or a postal questionnaire sent for completion and return.

### Phenotypes

In all assessments of the Isle of Wight birth cohort, eczema was defined as chronic or chronically relapsing, itchy dermatitis lasting more than 6 weeks with characteristic morphology and distribution [20], following Hanifin and Rajka criteria [21]. Since the 1-year and 2-year follow-up data on eczema were collected in a relatively small time window, we combined them for analytic purposes (reported as 1-or-2 years).

To determine allergic sensitization status, skin prick testing (SPT) at ages 1 and 2 years was performed on children with any symptoms of eczema, asthma, or rhinitis. We combined SPT results for ages 1 and 2 years, since they occurred within a short time period and will henceforth refer to this as SPT at 1-or-2 years. At 4, 10 and 18 years, regardless of symptoms, SPT was performed on most children attending the research center to a standard battery of common allergens (ALK-Albello, Horsholm, Denmark). Inhalant allergens tested were house dust mite, cat, dog, *Alternaria alternata*, *Cladosporium herbarium*, grass pollen mix, and tree pollen mix. Food allergens tested were cows' milk, soya, hens' egg, peanut and cod. Positive and negative controls were included. Allergic sensitisation was defined by having a SPT to at least one allergen test with mean wheal diameter of 3 mm greater than the negative control. Since allergic sensitization is a dynamic rather than a completely stable phenotype, we used the concurrent status and thus allowed the risk to change over time. In this manuscript we use the terms allergic sensitization and positive SPT interchangeably.

### *FLG* genotyping

We approached the participants in 1999 and ask them to provide written informed consent to genetic analyses, when blood samples were collected in the vast majority of children (n = 907). In 2006/2007, DNA samples were collected from an additional 304 children who did not provide the samples in 1999. DNA was extracted from blood or saliva samples from cohort subjects (n = 1,211). Five variants in the *FLG* gene that result in loss of function and are reported to be common in populations of European ancestry were selected for genotyping. DNA samples were interrogated using GoldenGate Genotyping Assays (Illumina, Inc, SanDiego, CA) on the BeadXpressVeracode platform (Illumina, Inc, SanDiego, CA) per Illumina's protocol. In brief, samples were fragmented and hybridized to the pool of allele-specific primer sets. Following an extension/ligation reaction the samples were then hybridized to the Veracode bead pool and processed on the BeadXpress reader. Data were analyzed using the genotyping module of the GenomeStudio Software package (Illumina, Inc, SanDiego, CA). DNA from each subject plus 37 replicate samples were analyzed for a total of 1,248 samples. The quality threshold for allele determination was set at a GenCall score >0.25 (scores ≤0.25 were “no calls”) with n = 1,227 samples (98.3%) retained for further analysis. Analysis of each locus included reclustering of genotyping data using our project data to define genotype cluster positions with additional manual reclustering to maximize both cluster separation and the 50th percentile of the distribution of the GenCall scores across all genotypes (50% GC score). Children were classified as having *FLG* loss-of-function defect if they carry the minor allele for at least one of the following *FLG* null variants: R501X, 2282del, or S3247X.

### Statistical analysis

The study represents a dynamic cohort; some children did not participate in one assessment but re-joined the next. For each follow-up, the 12-month (24-month for 1-or-2 years) period prevalence of eczema was stratified by allergic sensitization and *FLG* variants. Hence, the number of children with eczema was divided by the number of children in the respective stratum.

Since period prevalence and transitions of eczema and allergic sensitizations are not rare events (>10%), odds ratios for explanatory variables are likely to overestimate risk ratios [22]. To estimate risk ratios, we applied log-binomial regression using log link function describing the distribution of a binary variable (i.e. dichotomous outcomes). In this type of regression, the log-linear function describes the association between the mean number of “successes” in the binomial distribution on a log-scale and the explanatory variables. Thus, this approach enabled us to directly estimate risk ratios, which is the exponential of the estimated coefficients in the log-linear function. To assess long-term development in individual children, we had to consider that repeated measurements within one child represent correlated observations. The within-child effect was taken into consideration by employing a covariance matrix solved through an iterative estimating process based on a working correlation matrix. This is the idea of the generalized estimating equation (GEE) approach for estimating the parameters [23]. The appropriate working correlation matrix was determined by using the Akaike information criterion. Applying GEE analysis using an autoregressive working correlation matrix to take the within-child effect into account, we estimated marginal probabilities for the risk factors and their interaction using the GENMOD procedure in SAS 9.2 (SAS, Gary, NC, USA). For all GEE models we included age at follow-up as categorical variables (1-or-2, 4, 10, and 18 years) and gender as potential confounders. In this manuscript we use the terms repeated measurement analysis and longitudinal analysis interchangeably. A p-value<0.05 was taken to indicate statistical significance.

First for descriptive purposes, we analyzed concurrent associations between eczema and *FLG* variants, eczema and SPT, and *FLG* variants and SPT. Focusing on the overall cohort we calculated risk ratios (RR) using log-binomial regression. Within this approach, we then restricted the cohort to children with a negative SPT and estimated the association between eczema and *FLG* variants to determine the effect of *FLG* variants in the absence of allergic sensitization [24]. In contrast, to determine the effect of allergic sensitization in the absence of *FLG* variants, we focused on the cohort of children without *FLG* variants and evaluated that association between eczema and SPT. Then, to determine the combined effect of both risk factors, we compared the risk of eczema in children who have both risk factors (*FLG* variants and positive SPT) to the risk of children who have neither (no *FLG* variants and negative SPT). We also applied an alternate approach using the whole sample and treating *FLG* variants and allergic sensitization as main effects and estimated the combined effect through log-binomial model {RR = exp(β_1_×SPT+β_2_×*FLG* variants+β_3_×[SPT×*FLG* variants])} controlling for age and gender of the child. The interaction term (β_3_×[SPT×*FLG* variants]) was used to estimate the additional effect of two co-occurring risk factors on the health outcome above and beyond their individual effects. The term “combined effect” was used to describe the joint impact of two individual risk factors plus their interaction on the occurrence of the outcome. To estimate concurrent age-specific effects, we analyzed each examination separately. To evaluate whether there is an overall concurrent effect covering all ages, we used repeated measurements analyses.

**Figure 1:**
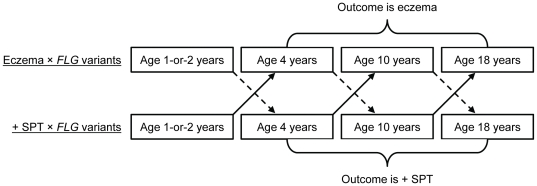
Graphical presentation of temporal (time-order) analyses. “+ SPT” refers to positive skin prick test. Between four follow-ups (ages 1-or-2, 4, 10, and 18 years) three transition periods (1-or-2 to 4, 4 to 10, and 10 to 18) were analyzed. The combined effect of eczema and FLG variants on the development of subsequent allergic sensitization (defined by positive SPT) was determined for the three transition periods separately and in repeated measurement analysis. Similarly, the combined effect of allergic sensitization and FLG variants on the development of subsequent eczema was determined. Dashed arrows refer to the combined effect of eczema and FLG variants on the development of + SPT. Solid arrows represent the combined effect of + SPT and FLG variants on the development of eczema.

To address temporality and to decipher whether allergic sensitization leads to eczema or vice versa, while accounting for the *FLG* variants effect, we structured the analysis into three transition periods (Figure 1). We have three transitions (1-or-2 to 4, 4 to 10, and 10 to 18 years) between four follow-ups (ages 1-or-2, 4, 10, and 18 years). For each transition period, at baseline children were without the outcome (allergic sensitization or eczema, respectively) but had either allergic sensitization or eczema (alternate risk factors). The value of the risk factors could change for each transition (time-dependent covariate), since eczema and SPT were conducted at each examination. This setting provides three repeated transition periods. In each we assessed the interaction between the risk factors (time-dependent) and *FLG* variants (time-independent) using log-binomial models. To address the overall effect of all three transition periods, we applied repeated measurements analyses. Hence, the alternate models were: 1) eczema model: *FLG* variants × preceding allergic sensitization → eczema; and 2) allergic sensitization model: *FLG* variants × preceding eczema → allergic sensitization.

## Results

A total of 1,377, 1,214, 1,359, and 1,307 children had available information on eczema diagnosis at ages 1-or-2, 4, 10, and 18 years, respectively. Skin prick tests (SPTs) were performed at ages 1-or-2 (n = 515, symptomatic children), 4 (n = 982), 10 (n = 1,036), and 18 (n = 853) years. Genotype information on the *FLG* variants was available for 1,150 children. No significant group differences were detected between the total cohort and the genotyped children (Table 1).

**Table 1:** Characteristics of total cohort and subpopulation of children with genotype information for *FLG* variants.

Characteristics	Total cohort% (n/total)	Genotypedsubpopulation*% (n/total)	*P*-value†
**Gender**			
Male	51.2 (786/1536)	49.5 (569/1150)	0.243
Female	48.8 (750/1536)	50.5 (581/1150)	0.243
**Eczema**			
At 1-or-2 years	14.2 (196/1377)	15.1 (162/1076)	0.421
At 4 years	11.9 (145/1214)	11.8 (119/1008)	0.926
At 10 years	13.7 (186/1359)	14.7 (164/1118)	0.346
At 18 years	12.3 (161/1307)	12.2 (132/1086)	0.884
**Allergic sensitization**			
At 1-or-2 years‡	20.6 (106/515)	21.9 (98/447)	0.489
At 4 years	19.6 (192/982)	20.4 (172/843)	0.557
At 10 years	26.9 (279/1036)	27.9 (266/954)	0.494
At 18 years	41.4 (353/853)	42.3 (336/794)	0.311

* Genotyped subpopulation represents the number of children with genotype information for R501X, 2282del and S3247X *FLG* variants.

† Two-sided one sample binomial tests were used to determine if statistical differences are present when comparing proportions for the genotyped subpopulation with their respective proportions in the total cohort.

‡ Skin prick tests were performed on symptomatic children (i.e. children with symptoms of eczema, asthma, or rhinitis) at age 1-or-2 years.

### *FLG* variants

Participants were genotyped for *FLG* variants R501X, 2282del4, S3247X, 3702delG, and R2447X. The polymorphic *FLG* variants were in Hardy-Weinberg equilibrium. Variants 3702delG and R2447X were not informative in our population due to minor allele frequencies <0.1% (Table S1). Therefore, *FLG* loss-of-function status was determined using R501X, 2282del4, and S3247X variants. Minor allele frequencies of R501X, 2282del4 and S3247X were 2.0%, 2.3% and 0.8%, respectively. The proportion of carriers (heterozygous) of *FLG* variants R501X, 2282del4 and S3247X were 4.1%, 4.6% and 1.6%, respectively, resulting in a combined carrier frequency of 10.3%. No individuals were homozygous for the minor allele of any *FLG* variants. One person had a compound heterozygous genotype for R501X and 2282del4, and this person was analyzed with the heterozygotes.

### Effect of *FLG* variants and allergic sensitization on eczema

The combined genotype of *FLG* variants was strongly associated with eczema at all time points except at 10 years of age (Table 2). This finding is further supported by repeated measurement analysis (age 1-or-2 to 18 years), which showed an overall effect of *FLG* variants on eczema ([Risk Ratio] RR = 1.56; 95% CI: 1.17–2.08). Likewise, allergic sensitization was associated with eczema at 1-or-2, 4, 10, and 18 years of age, with the strongest association observed at age 4 years (RR = 3.05; 95% CI: 2.21–4.2; Table 2).

**Table 2:** Risk ratios of *FLG* variants and allergic sensitization on the occurrence of concurrent eczema in the course of childhood and adolescence.

	*FLG* variants‡			Allergic sensitization	
% (n/total)	WT		LOF	Negative	Positive
**Eczema at 1-or-2**	14.1 (136/964)		23.2 (26/112)	29.6 (121/409)	56.6 (60/106)
**RR (95% CI)** *	1.00		1.64 (1.13–2.38)	1.00	1.94 (1.55–2.42)
***P*** **-value**			0.009		<0.001
**Eczema at 4**	10.8 (97/902)		20.8 (22/106)	9.0 (71/788)	27.5 (53/193)
**RR (95% CI)** *	1.00		1.92 (1.27–2.91)	1.00	3.05 (2.21–4.2)
***P*** **-value**			0.002		<0.001
**Eczema at 10**	14.1 (142/1004)		19.3 (22/114)	13.0 (98/757)	21.9 (61/279)
**RR (95% CI)** *	1.00		1.37 (0.91–2.05)	1.00	1.71 (1.28–2.29)
***P*** **-value**			0.129		<0.001
**Eczema at 18**	11.5 (112/975)		18.0 (20/111)	9.8 (49/500)	15.9 (56/353)
**RR (95% CI)** *	1.00		1.6 (1.04–2.46)	1.00	1.73 (1.21–2.48)
***P*** **-value**			0.031		0.003
**Repeated measurement analysis**					
**Eczema**		12.7 (487/3845)	20.3 (90/443)	13.8 (339/2454)	24.7 (230/931)
**RR (95% CI)** †		1.00	1.56 (1.17–2.08)	1.00	1.67 (1.41–1.97)
***P*** **-value**			0.003		<0.001

RR: Risk ratio; CI: Confidence interval; WT: Wild-type; LOF: Loss-of-function.

* Association adjusted for gender.

† Association adjusted for gender and age at follow-up.

‡ Combined genotypes of R501X, 2282del and S3247X variants; WT refers to individuals with wild-type genotypes for all three variants; LOF refers to individuals with a minor allele for at least one of the three variants.

### Effect of *FLG* variants on allergic sensitization

Association analyses between *FLG* variants and allergic sensitization were conducted at 1-or-2, 4, 10, and 18 years of age, and to determine the overall association a repeated measurement approach was applied. *FLG* variants increased the risk of allergic sensitization at age 1-or-2 years (RR = 1.57; 95% CI: 1.02–2.4; Table S2) and at age 10 years (RR = 1.53; 95% CI: 1.18–1.99; Table S2). At other ages (4 and 18 years) and in the longitudinal analysis the associations were weaker.

### Single and combined associations of *FLG* variants and allergic sensitization with eczema

To examine the individual and combined effect of *FLG* variants and allergic sensitization on the concurrent risk of eczema, we used stratified analyses. Associations between *FLG* variants and eczema were conducted among a sub-group of children who tested negative in SPT assessments. This stratification allowed us to determine the effect of *FLG* variants on the development of eczema independent of allergic sensitization. *FLG* variants did not increase the risk of eczema in the sub-group of non-allergic children at any age or in the longitudinal analysis (RR = 1.12; 95% CI: 0.77–1.62; Table 3). Next, to determine the effect of allergic sensitization in the absence of *FLG* variants, we evaluated the association between positive SPT and concurrent eczema among children who do not carry *FLG* variants. At 1-or-2, 4, and 10 years (not at 18 years) of age a positive SPT increased risk of eczema (longitudinal analysis, RR = 1.48; 95% CI: 1.22–1.8) among children without *FLG* variants. Then we assessed the combined effect of *FLG* variants and allergic sensitization by comparing the risk of eczema in children with both *FLG* variants and positive SPT to the risk of eczema among children without *FLG* variants and who had a negative SPT. The combined effect of *FLG* variants and allergic sensitization was significant at all ages (repeated measurement analysis: RR = 2.82; 95% CI: 2.15–3.69) with a maximum effect at 4 years of age (RR = 8.17; 95% CI: 5.55–12.02; Table 3). Finally, alternative to stratification, we applied a log-binomial regression model to estimate the interaction between *FLG* variants and allergic sensitization using repeated measurements. This approach, controlling for the main effect of *FLG* variants and SPT, showed that *FLG* variants and positive SPT have an increased combined effect on the concurrent risk of eczema (RR = 2.51; 95% CI: 1.86–3.38; Table 3). Thus, stratification and modelling approaches yielded comparable results.

**Table 3:** Risk ratios of *FLG* variants, allergic sensitization, and their combined effect on the occurrence of concurrent eczema at different ages.

	*FLG* variants effect* in non-atopic children		Allergic sensitization effect† in children without *FLG* variants		Combined effect‡ of *FLG* variants and allergic sensitization	
% (n/total)	WT	LOF	SPT −	SPT +	WT & SPT −	LOF & SPT +
**Eczema at 1-or-2**	28.0 (87/311)	31.6 (12/38)	28.0 (87/311)	53.8 (43/80)	28.0 (87/311)	72.2 (13/18)
**RR (95% CI)** ¶	1.00	1.13 (0.68–1.86)	1.00	1.93 (1.47–2.52)	1.00	2.67 (1.93–3.69)
***P*** **-value**		0.638		<0.001		<0.001
**Eczema at 4**	8.3 (50/605)	9.0 (6/67)	8.3 (50/605)	20.6 (30/146)	8.3 (50/605)	64 (16/25)
**RR (95% CI)** ¶	1.00	1.08 (0.48–2.43)	1.00	2.49 (1.65–3.77)	1.00	8.17 (5.55–12.02)
***P*** **-value**		0.849		<0.001		<0.001
**Eczema at 10**	13.3 (84/632)	12.5 (7/56)	13.3 (84/632)	19.8 (45/227)	13.3 (84/632)	30.8 (12/39)
**RR (95% CI)** ¶	1.00	0.94 (0.46–1.94)	1.00	1.5 (1.08–2.09)	1.00	2.52 (1.49–4.23)
***P*** **-value**		0.869		0.016		<0.001
**Eczema at 18**	10.1 (42/416)	7.1 (3/42)	10.1 (42/416)	13.7 (40/293)	10.1 (42/416)	25.6 (11/43)
**RR (95% CI)** ¶	1.00	0.73 (0.24–2.24)	1.00	1.46 (0.97–2.19)	1.00	3.09 (1.78–5.37)
***P*** **-value**		0.584		0.069		<0.001
**Repeated measurement analysis**						
**Eczema**	13.4 (263/1964)	13.8 (28/203)	13.4 (263/1964)	21.2 (158/746)	13.4 (263/1964)	41.6 (52/125)
**RR (95% CI)** $	1.00	1.12 (0.77–1.62)	1.00	1.48 (1.22–1.8)	1.00	2.82 (2.15–3.69)
***P*** **-value**		0.571		<0.001		<0.001

Repeated measurement analysis (modeling approach)						
Variable	Coefficient	RR (95% CI)	*P*-value			
*FLG* variants	0.08	1.09 (0.74–1.59)	0.675			
Allergic sensitization	0.41	1.51 (1.24–1.84)	<0.001			
Interaction term	0.43	1.53 (0.94–2.49)	0.089			
**Combined effect** #	0.92	2.51 (1.86–3.38)	<0.001			

RR: Risk ratio; CI: Confidence interval; WT: Wild-type; LOF: Loss-of-function; SPT −: Negative skin prick test; SPT +: Positive skin prick test.

Combined genotypes of R501X, 2282del and S3247X variants; WT refers to individuals with wild-type genotypes for all three variants; LOF refers to individuals with a minor allele for at least one of the three variants.

* All children are non-allergic (defined by negative skin prick test at the concurrent eczema assessment).

† All children are wild type for *FLG* null variants.

‡ Children with both positive skin prick test and *FLG* LOF variants were compared to children with negative skin prick test and wild-type *FLG*.

¶ Association adjusted for gender.

$ Association adjusted for gender and age at follow-up.

# The combined effect RR was estimated as: RR = exp(0.08+0.41+0.43).

### Association analyses accounting for temporal sequence of events

To address temporality of allergic sensitization and eczema we analyzed three transition periods (1-or-2 to 4, 4 to 10, and 10 to 18 years) between four follow-ups (ages 1-or-2, 4, 10, and 18). At the baseline of the transition period, children were without the outcome but had developed eczema in the next exam: 6.2%, 9.4%, and 6.7%, respectively, between 1-or-2 and 4 years, 4 and 10 years, and 10 and 18 years, or had developed allergic sensitization: 14.2%, 14.8%, and 23.1%, respectively (Table 4). The combined effect of allergic sensitization in the preceding exam and *FLG* variants on the development of eczema (eczema model) showed a 2.92-fold increased risk (95% CI: 1.47–5.77) in the longitudinal analysis. Alternatively, we tested whether the combined effect of eczema in the preceding exam and *FLG* variants affected the development of positive SPT (allergic sensitization model), but did not find a significant association in the repeated measurements analysis.

**Table 4:** Risk ratios of *FLG* variants interacting with allergic sensitization and eczema on the subsequent occurrence of eczema (Eczema model) and allergic sensitization (Allergic sensitization model).

Eczema model*			Allergic sensitization model†		
Transition of eczema % (n/total)‡			Transition of allergic sensitization % (n/total)‡		
Transition period (age in years)			Transition period (age in years)		
1-or-2 to 4	4 to 10	10 to 18	1-or-2 to 4	4 to 10	10 to 18
6.2 (18/292)	9.4 (69/732)	6.7 (51/761)	14.2 (44/310)	14.8 (90/607)	23.1 (118/512)

Repeated measurement analysis				Repeated measurement analysis			
Variable	Coefficient	RR(95% CI)	*P*-value	Variable	Coefficient	RR(95% CI)	*P*-value
Allergic sensitization	−0.36	0.7(0.42–1.15)	0.157	Eczema	0.15	1.16(0.84–1.61)	0.367
*FLG* variants	0.04	1.04(0.57–1.89)	0.896	*FLG* variants	0.1	1.11(0.72–1.7)	0.651
Interaction term	1.39	4.02(1.49–10.85)	0.006	Interaction term	0.31	1.36(0.53–3.5)	0.519
**Combined effect** ¶	1.07	2.92(1.47–5.77)	0.002	**Combined effect** $	0.56	1.75(0.79–3.85)	0.166

RR: Risk ratio; CI: Confidence interval.

* This log-binomial model tests whether allergic sensitization, *FLG* variants and their interaction predict the transition of eczema (change is disease status from eczema-free to eczema in three consecutive study assessments). Also, gender and age at follow-up were included in the model as covariates.

† This log-binomial model tests whether eczema, *FLG* variants and their interaction predict the transition of allergic sensitization (change in status from having negative skin prick test to positive skin prick test in three consecutive study assessments). Also, gender and age at follow-up were included in the model as covariates.

‡ Refers to the percent of children who were eczema-free or non-atopic at the initial assessment of the transition period and developed eczema or allergic sensitization at the second assessment of the same transition period.

¶ RR of combined effect of allergic sensitization and *FLG* variants on development of eczema was calculated as: RR = exp(−0.36+0.04+1.39).

$ RR of combined effect of eczema and *FLG* variants on development of allergic sensitization was calculated as: RR = exp(0.15+0.1+0.31).

**Figure 2:**
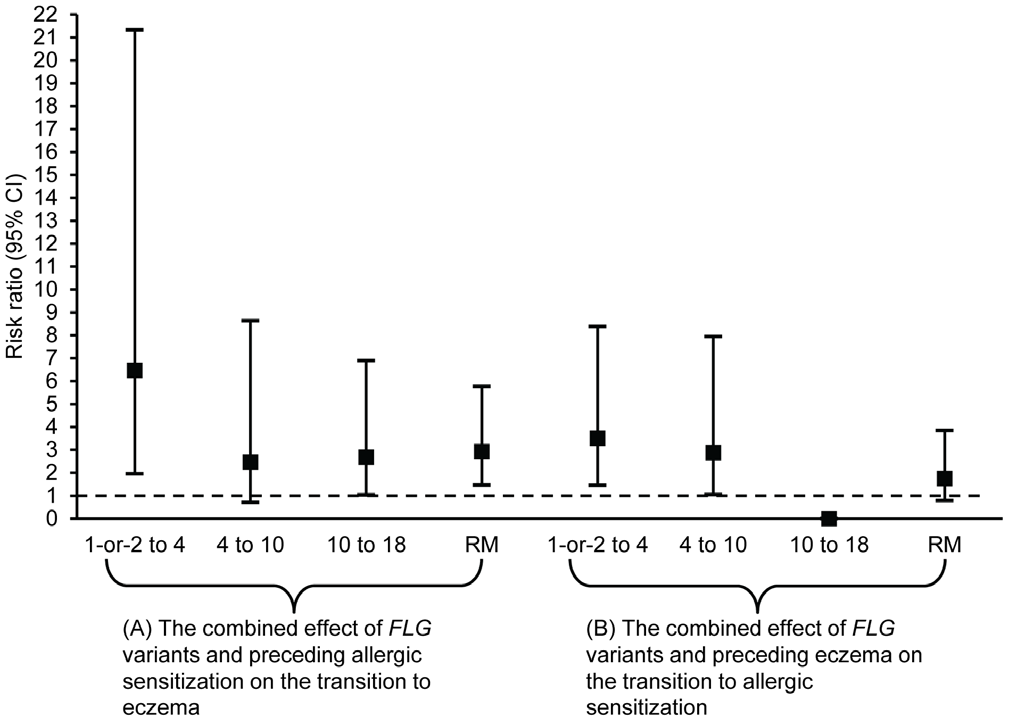
Overall and time-specific risk ratios in the temporal (time-order) analyses. (A) Risks ratios associated with the combined effect of *FLG* variants and allergic sensitization on the development of subsequent eczema for three transition periods and repeated measurements. (B) Risk ratios associated with the combined effect of *FLG* variants and eczema on the development of subsequent allergic sensitization for three transition periods and repeated measurements. “RM” refers to repeated measurements.

To decipher whether time (transition-period) specific effects are present in the analysis of the temporal sequence, we determined the risk associated with the combined effect for each transition separately. The combined effect of *FLG* variants and allergic sensitization statistically significantly increased the risk of subsequent eczema for the transition periods 1-or-2 to 4 years (RR = 6.47; 95% CI: 1.96–21.33, p-value = 0.002; Figure 2A) and 10 to 18 years (RR = 2.68; 95% CI: 1.04–6.9, p-value = 0.041), which was further supported by repeated measurements analysis that suggested the overall effect of all transition periods is statistically significant (Table 4). In contrast, the combined effect of *FLG* variants and eczema statistically significantly increased the risk of allergic sensitization only for the transition periods 1-or-2 to 4 years (RR = 3.5; 95% CI: 1.46–8.39, p-value = 0.005) and 4 to 10 years (RR = 2.88; 95% CI: 1.05–7.95, p-value = 0.041; Figure 2B). The overall effect of the three transitions periods for this model (allergic sensitization model) was not statistically significant (Table 4).

## Discussion

Using data of a large follow-up study from infancy to age 18 years, our study explores two assertions: first, the combined occurrence of eczema and *FLG* variants leads to allergic sensitization, second, the combined effect of allergic sensitization and *FLG* variants leads to eczema. We applied two explanatory models, a concurrent and a delayed effect (time order) model (Figure 1), and investigated both the overall effect (repeated measurements) of eczema and allergic sensitization and period-specific effects (ages 1-or-2, 4, 10 and 18 years). In the repeated measurement analyses of concurrent effects, *FLG* variants, in the absence of a positive SPT, did not significantly increase the risk of the concurrent eczema (RR = 1.12). In contrast, among individuals without *FLG* variants, allergic sensitization was associated with concurrent risk of eczema (RR = 1.48). In the presence of both *FLG* variants and allergic sensitization (combined effect), a considerable increase in the risk ratio was observed (RR = 2.82). Focussing on the temporal sequence in repeated measurement analyses, we found that among eczema-free children, a positive SPT combined with *FLG* variants showed a significant interaction (combined effect RR = 2.92) on the occurrence of eczema in subsequent follow-up examinations up to 18 years of age. In contrast, in non-sensitized children the combined effect of eczema and *FLG* variants posed no overall increased risk for subsequent allergic sensitization, but only for the transition periods 1-or-2 to 4 years and 4 to 10 years.

Other birth cohort studies, the Copenhagen Study on Asthma in Childhood (COPSAC), the Manchester Asthma and Allergy Study (MAAS), the Prevention and Incidence of Asthma and Mite Allergy (PIAMA) in the Netherland, and the Avon Longitudinal Study of Parents and Children (ALSPAC), have ascertained *FLG* variants and eczema longitudinally up to the age of 5, 8, and 11 years, respectively, with several repeated measurements [13], [25], [26]. However, the temporal sequence of eczema and allergic sensitization in interaction with *FLG* variants was not tested. Nonetheless, when comparing the results of our concurrent models with these studies, the magnitude of the associations between *FLG* variants and eczema is comparable. In our cohort, at 4 years of age *FLG* variants increased the risk of eczema by approximately 2-fold (RR = 1.92; 95% CI: 1.27–2.91). Similarly, results from the COPSAC cohort study demonstrated that *FLG* variants increase the risk of eczema during the first five years of life by almost the same amount (OR = 1.75; 95% CI: 1.29–2.37) [27]. Furthermore, results from the PIAMA cohort study showed that *FLG* variants were associated with eczema during the first eight years of life (OR = 2.0; 95% CI: 1.4–2.8) [25]. A slightly higher risk of eczema due to *FLG* variants during the first 11 years of life (OR = 2.46; 95% CI: 2.02–2.99) was reported by the ALSPAC cohort study [13].

A report of the German Multicentre Allergy Study (MAS) cohort show an interaction effect of *FLG* variants and food sensitization (RR = 4.07; 95% CI: 2.94–5.65) on the development of concurrent “eczema-associated asthma” from birth to 13 years of age [28]. In our study we investigated the combined effects of *FLG* variants and allergic sensitization (instead of food sensitization only) on the development of eczema (not “eczema-associated asthma”). Using repeated measurements from age 1-or-2 to age 18, we found that allergic sensitization interacts with *FLG* variants and increase the risk of concurrent eczema (RR = 2.82).

Without considering *FLG* variants, previous longitudinal investigations showed inconsistent results as to whether manifestation of eczema occurs before or after allergic sensitization development [18]. A report based on Melbourne Atopy Cohort Study (MACS, investigating the period from 6 months to 7 years of age) suggested that allergic sensitization may precede eczema development or eczema develops before allergic sensitization [29]. In contrast, results from the Childhood Asthma Prevention Study (CAPS) cohort in Sydney showed that eczema preceded and increased the risk of subsequent allergic sensitization between ages 18 months to 5 years [30]. Both studies did not account for the effect of *FLG* variants and did not cover the period of adolescence. When investigating the temporal sequence of events in relation to *FLG* variants effect, we found that both directions coexist. The combined effect of *FLG* variants and eczema leading to later allergic sensitization was only seen in the first decade of life. This finding speaks in favour of the model proposed by Cork et al., suggesting that the skin barrier is less developed at younger ages, which might explain the increased risk of allergic sensitization associated with *FLG* variants and eczema only in infants and young children [5]. For the transition period 10 to 18 years, the combined effect of *FLG* variants and eczema had no effect on the development of subsequent allergic sensitization. This observation is further supported by a previous study which showed that the majority of children carrying *FLG* variants outgrow eczema by 12 years of age [13]. Hence, in the transition from 10 to 18 years, the eczema × *FLG* variants interaction may no longer add to the risk of allergic sensitization. Overall, compared to the combined effect of eczema and *FLG* variants on allergic sensitization, the combined effect of *FLG* variants and allergic sensitization leading to eczema was stronger, particularly in early childhood, and covered the first two decades of life (Figure 2). This novel finding suggests strongly that allergic sensitization is not an epiphenomenon of eczema or its sequel, but a risk factor for subsequent eczema.

The longitudinal design of the Isle of Wight birth cohort study that covered the first 18 years of life is a major strength of our study. Throughout all assessments, the proportion of participation remained high, which ruled-out a major bias due to loss-to-follow up. Moreover, SPTs were repeatedly performed, thus allowing us to treat allergic sensitization as a time-dependant covariate. The prevalence of eczema and allergic sensitization did not differ between the total cohort and the sample that had available *FLG* variants genotypes (Table 1). Thus, there is no indication of a selection bias. Misclassification of eczema cases is minimal since a high proportion of subjects showed typical manifestation of eczema in usual locations (antecubital or popliteal fossae, ankles, face or neck for 97% at 1 year, 91% at 2 years, 75% at 4 years, 86% at 10 years and 76% at 18 years) [31]. In addition, the prevalence of eczema in our cohort (13.7% at age 10 years and 12.3% at age 18 years) is comparable to results from the International Study of Asthma and Allergies in Childhood aged 13–14 years in the United Kingdom (UK) (14.7% phase one and 10.6% phase three) and other studies conducted in the UK [32], [33], [34]. The proportion of *FLG* variants carriers in our study population (10.3%) is similar to the estimated heterozygous carries (10%) for individuals of European ancestry [12]. In line with previous studies, individuals homozygous for the minor allele of *FLG* variants were rarely identified due to the low minor allele frequencies of these variants [13], [25], [35].

A limitation of this study is that we have information on SPTs for the exams at age 1 and 2 years only for symptomatic children. However, there is no suspicion that the inclusion of the SPT at age 1 and 2 resulted in a selection bias: First, in both the eczema and the control children at age 1-or-2 the proportion of positive SPT is not higher than at age 4 when all available children were skin prick tested (21.9 vs. 20.4, Table 1). Second, there is no increased risk ratio of allergic sensitization at age 1-or-2 for eczema compare to other ages (Table 2). Statistical power could be another potential limitation due to the low sample sizes in some of the analyzed subgroups. However, statistical significance was demonstrated for most of the clinically important effects (i.e. associations with RR considerably higher than the null). Therefore, we believe that our study was not underpowered to detect statistically significant and clinically important associations.

Our results suggest that a defective skin barrier and altered immune responses work synergistically to increase susceptibility to eczema, a concept recently proposed by Biagini Myers et al. [36]. Null variants in *FLG* result in the formation of a disrupted skin barrier that predisposes to higher risk of eczema due to higher penetration of environmental factors (outside-inside barrier) and excessive water loss (inside-outside barrier) [17]. Our findings, that allergic sensitization is associated with eczema even in the absence of *FLG* variants, further support the concept that penetration of allergens leading to manifestation of eczema may also be related to other factors that regulate the integrity of the skin barrier. Therefore, factors regulating the immune response as well as genes responsible for the formation of functional skin barrier, may play a role in the pathophysiology of eczema [37].

A recent study showed that the skin barrier is compromised even in non-*FLG* variants carriers, supporting the notion that other factors may be important for the formation of a functional skin barrier [38]. Recent reports suggested that a single nucleotide polymorphism within the *HRNR* (hornerin) gene, located 78 kb away from *FLG*, conferred susceptibility to eczema [39], [40]. Hence comprehensive tests of genetic variants in the epidermal differentiation complex will improve the sensitivity of identifying susceptible children. Improved knowledge of skin susceptibility factors would then enhance randomization of susceptible groups to test treatment effects of possible skin treatments.

In conclusion, no prior study has investigated the long-term development of eczema and allergic sensitization with respect to interactions with *FLG* variants. The results of our longitudinal study covering the first 18 years of life corroborate prior findings that a higher risk of eczema is associated with *FLG* variants. For the first time, taking the time-order into account, we estimated the risk ratio of interaction of *FLG* variants with allergic sensitization and eczema on later occurrence of eczema or allergic sensitization, respectively. We demonstrated that both directions coexist; however, the interaction between allergic sensitization and *FLG* variants on subsequent eczema was more important: First, this interaction was detected in both childhood and adolescence, whereas the effect of the *FLG* variants × eczema interaction was only seen up to the age of 10 years. Second, the interaction *FLG* variants × allergic sensitization was stronger particularly in early childhood. The findings suggest that improvements of the skin barrier, for example by using barrier creams or anti-inflammatory treatments, at an early age may reduce the risk of eczema associated with the combination of *FLG* variants and allergic sensitization.

## Supporting Information

Table S1 **Summary of** ***FLG*** **variants genotype data.**
(PDF)

Table S2 **Risk ratios of** ***FLG*** **variants for allergic sensitization in the course of childhood and adolescence.**
(PDF)
