# Supplementary material for: Interplay of Filaggrin Loss-of-Function Variants, Allergic Sensitization, and Eczema in a Longitudinal Study Covering Infancy to 18 Years of Age
Source: PLoS One. 2012 Mar 5;7(3):e32721. doi: 10.1371/journal.pone.0032721 (PMC3293849; doi:10.1371/journal.pone.0032721)
Supplement: Table S1 — Summary of FLG variants genotype data. (PDF) [file pone.0032721.s001.pdf]

Table S1. Summary of *FLG* variants genotype data

Variant	Chromosome position	Wild-type genotype frequency	Heterozygous genotype frequency	Minor allele frequency	HWE P-value
R501X	150552485	0.9595	0.0405	0.02	0.48
2282del4	150551701	0.9538	0.0462	0.023	0.42
S3247X	150550284	0.9845	0.0155	0.008	0.79
3702delG	150546647	0.999	0.001	0.0005	0.99
R2447X	150544246	1	0	0	NE

HWE: Hardy-Weinberg Equilibrium; NE: Not Estimable.
No individuals were homozygous for the minor allele for any *FLG* null variant.
P-value is testing the hypothesis that variants are in HWE.
