# Supplementary material for: Interplay of Filaggrin Loss-of-Function Variants, Allergic Sensitization, and Eczema in a Longitudinal Study Covering Infancy to 18 Years of Age
Source: PLoS One. 2012 Mar 5;7(3):e32721. doi: 10.1371/journal.pone.0032721 (PMC3293849; doi:10.1371/journal.pone.0032721)
Supplement: Table S2 — Risk ratios of FLG variants for allergic sensitization in the course of childhood and adolescence. (PDF) [file pone.0032721.s002.pdf]

Table S2. Risk ratios of *FLG* variants for allergic sensitization in the course of childhood and adolescence

	***FLG* variants**‡	
**% (n/total)**	**WT**	**LOF**
**Sensitization at 1-or-2**	20.5 (80/391)	32.1 (18/56)
**RR (95% CI)***	1.00	1.57 (1.02-2.4)
***P*-value**		0.04
**Sensitization at 4**	19.4 (146/751)	27.2 (25/92)
**RR (95% CI)***	1.00	1.38 (0.96-1.99)
***P*-value**		0.089
**Sensitization at 10**	26.4 (227/859)	41.1 (39/95)
**RR (95% CI)***	1.00	1.53 (1.18-1.99)
***P*-value**		0.002
**Sensitization at 18**	41.3 (293/709)	50.6 (43/85)
**RR (95% CI)***	1.00	1.18 (0.95-1.48)
***P*-value**		0.138
**Repeated measurement analysis**		
**Sensitization**	27.5 (746/2710)	38.1 (125/328)
**RR (95% CI)**†	1.00	1.20 (0.98-1.48)
***P*-value**		0.081

RR: Risk Ratio; CI: Confidence interval; WT: Wild-type; LOF: Loss-of-function.

* Association adjusted for gender.

† Association adjusted for gender and age at follow-up.

‡ Combined genotypes of R501X, 2282del and S3247X variants; WT refers to individuals with wild-type genotypes for all three variants; LOF refers to individuals with a minor allele for at least one of the three variants.
